# Neem oil nanoemulsions: characterisation and antioxidant activity

**DOI:** 10.1080/14756366.2017.1378190

**Published:** 2017-10-02

**Authors:** Federica Rinaldi, Patrizia Nadia Hanieh, Catia Longhi, Simone Carradori, Daniela Secci, Gokhan Zengin, Maria Grazia Ammendolia, Elena Mattia, Elena Del Favero, Carlotta Marianecci, Maria Carafa

**Affiliations:** a Fondazione Istituto Italiano di Tecnologia, Center for Life Nano Science@Sapienza, Rome, Italy;; b Dipartimento di Chimica e Tecnologie del Farmaco, “Sapienza” University of Rome, Rome, Italy;; c Dipartimento di Sanità pubblica e Malattie infettive, “Sapienza” University of Rome, Rome, Italy;; d Dipartimento di Farmacia, “G. d’Annunzio” University of Chieti-Pescara, Chieti, Italy;; e Department of Biology, Selçuk Üniversitesi Alaeddin Keykubat Yerleşkesi, Konya, Turkey;; f Centro nazionale per le tecnologie innovative in sanità pubblica, Istituto Superiore di Sanità, Rome, Italy;; g Dipartimento di Biotecnologie Mediche e Medicina Traslazionale, University of Milan, Segrate, Italy

**Keywords:** Neem oil, nanoemulsions, physicochemical characterisation, antioxidant activity, cell interaction studies

## Abstract

The aim of the present work is to develop nanoemulsions (NEs), nanosized emulsions, manufactured for improving the delivery of active pharmaceutical ingredients. In particular, nanoemulsions composed of Neem seed oil, contain rich bioactive components, and Tween 20 as nonionic surfactant were prepared. A mean droplet size ranging from 10 to 100 nm was obtained by modulating the oil/surfactant ratio. Physicochemical characterisation was carried out evaluating size, ζ-potential, microviscosity, polarity and turbidity of the external shell and morphology, along with stability in simulated cerebrospinal fluid (CSF), activity of Neem oil alone and in NEs, HEp-2 cell interaction and cytotoxicity studies. This study confirms the formation of NEs by Tween 20 and Neem oil at different weight ratios with small and homogenous dimensions. The antioxidant activity of Neem oil alone and in NEs was comparable, whereas its cytotoxicity was strongly reduced when loaded in NEs after interaction with HEp-2 cells.

## Graphical abstract

**Figure:**
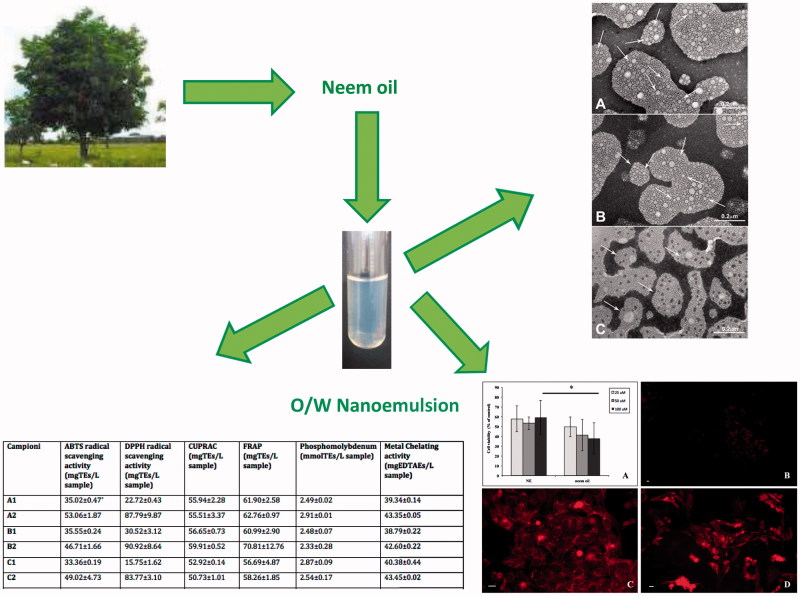

## Introduction

According to the European Commission definition, nanotechnology is a branch of engineering devoted to designing, producing and using structures and devices having one or more dimensions of about 100 nanometres or less. Nanoscience and nanotechnology are the study and application of extremely small things and can be used across all the other science fields, such as chemistry, biology, physics, materials science, engineering and in pharmaceutical sector. New or extensively used drugs can be better formulated if loaded in specific nanocarriers in order to overcome problems such as low solubility, large biodistribution, low bioavailability, adverse effects, high costs and so on. Along the diverse nanocarriers that are widely used in pharmaceutics, nanoemulsions have attracted great attention in drug delivery and pharmacotherapy. In particular, nanoemulsions act as an excellent and versatile vehicle for poorly aqueous soluble drugs, which are otherwise difficult to formulate in conventional dosage forms. Nanoemulsions are submicron emulsions composed of generally recognized as safe (GRAS) grade excipients approved by the United States Food and Drug Administration (FDA) and with the possibility to use different oils that can modulate the drug activity. Nanoemulsions are easily produced in large quantities by high shear stress or mechanical extrusion process and can be engineered with specific attributes such as size, surface charge, prolonged blood circulation and targeting properties. Nanoemulsions can be prepared in order to tune their physicochemical properties to be applied in therapy, diagnostics or theranostics.

The size of the droplets varies depending on the drug particles, mechanical energy, composition and relative amount of the surfactants^1^. Nanoemulsions are also known as miniemulsions, fine-dispersed emulsions, submicron emulsions, etc., which can be either oil in water (O/W) or water in oil (W/O) emulsion. The amount of oil in O/W nanoemulsions may vary but generally is within 5–20% w/w. Sometimes a mixture of oils may be used to improve drug solubilisation in the oil phase. A co-surfactant or a co-solvent may be used in addition to the surfactant to facilitate the stabilisation process^2^ ^,^ ^3^. The significant property that differentiates the nanoemulsions from other emulsion systems is that a nanoemulsion shows a different pattern in physical and rheological properties with decreasing droplet size. Nanoemulsion is much more stable than other emulsion systems and is more translucent compared to microemulsions^4^. Few nanoemulsions have been manufactured commercially in oral, topical, ophthalmic and even in intravenous (IV) drug delivery system. IV administration of nanoemulsions requires biodegradable surfactants. Numerous researches are presently being carried out in order to manufacture nanoemulsions from different classes of drugs for a variety of purposes. The use of nanoemulsions spreads from antibiotic therapy, atherosclerosis treatment, transdermal drug delivery and ophthalmic application to as far as cancer therapy, vaccine delivery, etc^5–7^.

One of the most appealing challenges in nanotechnology is to find an opportune strategy to bypass blood–brain barrier (BBB) and to reach the central nervous system (CNS). Among the numerous approaches proposed, the direct nose-to-brain drug delivery is a potential strategy to overcome the obstacles presented by the BBB. Intranasal delivery bypasses the BBB to target CNS, reducing systemic exposure of drug, thereby reducing the systemic side effects. It is an attractive option of drug delivery due to its noninvasiveness^8–10^.

Moreover, several researches suggest that the “nose-to-brain” route is one of the most important developments of pharmaceutical research in brain treatment, including the following: (i) the potential to avoid gastrointestinal (GI) and hepatic first-pass metabolism; (ii) the possibility of delivering drugs not suitable for oral administration, such as peptides and proteins; and (iii) most importantly, the transport of exogenous material directly from the nasal cavity to the brain, thus bypassing the BBB^11^.

It is still unknown whether the drug is being released from the carrier system in the nasal cavity and transported to CNS, or the carrier system is transported along olfactory and/or trigeminal nerve pathways into the CNS where the drug is released. Thus, more basic research is required to determine the possible transport pathway of therapeutic carrier to the CNS and their further fate into the biological system^8^.

In addition to the vesicular systems, widely used in nasal administration, other carriers are able to encapsulate drug and to perform intranasal delivery to CNS, such as cyclodextrins, micro- and nanoemulsions and nanoparticles^11^.

Nanoemulsions, by virtue of their lipophilic nature and low globule size, are widely explored as a delivery system to enhance uptake across the nasal mucosa. The possibility of using mucoadhesive agents such as polyelectrolyte polymers can prolong the nasal mucosa interaction, providing an extended delivery of the drug to the olfactory region and henceforth to the brain^8^.

The aim of this research study was to prepare and characterise a functional O/W nanoemulsion by using Neem oil. Neem oil is a deep yellow extract from *Azadirachta indica* A. Juss. (Meliaceae) seeds widely used in India and in other South-East countries as versatile medicinal product for several diseases, as pesticide/insecticide, and in cosmetics due to its low toxicity in *in vivo* studies^12^. These pharmaceutical properties are correlated with the content of limonoids (e.g. azadirachtin) as the most bioactive metabolites^13^. In this study, we made an effort to prepare nanoemulsions from Neem seed oil. We used Tween 20 as a nonionic surfactant because nonionic surfactants are known to be less affected by pH^14^ ^,^ ^15^. The aim was to prepare NEs with the possibility of combining, in a future study, the pharmacological properties of Neem oil and those of the therapeutic agent targeted by nanoemulsion.

## Materials and methods

### Materials

Neem oil was purchased by Neem Italia (Moniga del Garda (BS), Italy) and is characterised by an ECOCERT certificate (Biocert Italia IT013BC041 – ICEA 264BC001). Tween 20 (Tw20), Hepes salt {*N*-(2-hydroxyethyl) piperazine-*N*-(2-ethanesulphonic acid)} and pyrene were Sigma-Aldrich products (Sigma-Aldrich, Milan, Italy). Distilled water (Ecological Product System s.r.l., Rome, Italy) was used for all experiments. All other products and reagents were of analytical grade.

### Nanoemulsion preparation

Nanoemulsions (NEs) were prepared using different amounts of Tw20 and Neem oil (Table 1) in different weight ratios. Tw20 concentration in the samples was always remarkably above CMC (0.048 mM in water, at 20 °C).

**Table 1.** Sample compositions, size, ζ-potential, PDI values and lipophilic shell features (polarity and microviscosity) of NEs.

Samples	Medium	Noil (%w/w)	Tw20 (%w/w)	HD (nm) ± SD	ζ-Pot (mV) ± SD	PDI ± SD	Polarity (AU) ± SD	MicroV (AU) ± SD
A1	Water	33.3	66.6	30.98 ± 0.49	−7.29 ± 1.54	0.20 ± 0.01	0.97 ± 0.01	1.18 ± 0.02
A2	Hepes			30.47 ± 0.28	−16.80 ± 1.22	0.23 ± 0.01	0.94 ± 0.02	1.19 ± 0.01
B1	Water	20.0	80.0	21.73 ± 0.30	−6.35 ± 1.87	0.22 ± 0.01	0.97 ± 0.01	1.22 ± 0.03
B2	Hepes			22.62 ± 0.23	−13.80 ± 1.40	0.24 ± 0.01	0.97 ± 0.01	1.22 ± 0.01
C1	Water	12.4	83.6	15.42 ± 0.11	−5.45 ± 0.21	0.18 ± 0.01	0.97 ± 0.01	1.21 ± 0.02
C2	Hepes			16.76 ± 0.27	−15.00 ± 1.79	0.22 ± 0.01	0.96 ± 0.01	1.21 ± 0.01

NE samples were prepared using two different aqueous media: water and Hepes buffer (10^−2 ^M pH 7.4).

Noil: Neem oil; Tw20: Tween 20; HD: hydrodynamic diameter; ζ-Pot: ζ-potential; MicroV: microviscosity.

Results are expressed as means ± SD (*n* = 3).

Where indicated, the fluorescent probe pyrene (4 mM) was added to the surfactant/oil mixture before NE preparation. The NEs were obtained by a simple preparation method, as previously reported^16^. Neem oil, Tw20 and distilled water, or Hepes buffer (10^−2 ^M, pH 7.4) where indicated, were used in the preparation of water-based (O/W) emulsion. The emulsions contained Neem oil and surfactant with ratios of 1:2, 1:4 and 1:7, respectively. To form the emulsion, the two phases were vortexed for about 5 min. Each emulsion with microscale droplets was sonicated for 20 min at 50 °C using a tapered microtip operating at 20 kHz at an amplitude of 18% (Vibracell-VCX 400, Sonics, Taunton, MA) to obtain NEs.

### Dynamic light scattering measurements

Dynamic light scattering (DLS) on a Malvern Zetasizer Nano ZS90 (Malvern Instruments Ltd., Worcestershire, United Kingdom) was used to measure the size and the ζ-potential of the NEs allowing to determine the mass distribution of particle size as well as the dispersed particle electrophoretic mobility. Obtained data represent means of the measurements of ζ-potential (mV) and of the hydrodynamic diameter (nm) for the NE droplets. It is important to notice that size distribution results are expressed as % of intensity of the colloidal dispersion. The polydispersity index (PDI) value was also determined as an evaluation of the breadth of the size distribution: a PDI value lower than 0.3 indicates a homogenous and monodisperse population.

### Lipophilic shell characterisation

The fluorescence experiments on NEs incorporating pyrene were carried out to evaluate the micropolarity of the lipophilic shell surrounding the oil droplet by a Perkin-Elmer LS55 spectrofluorometer. Using the pyrene probe, which is entrapped mainly in the lipophilic shell, it is possible to investigate the lateral distribution and the dynamics of membrane compounds. Pyrene is a spatially sensitive probe, displaying a peculiar band around 460 nm when two molecules are spatially proximal (excimer); it is worthy to notice that when the excimer fluorescence decreases, there is an increase in monomer fluorescence. The monomer and the excimer possess different fluorescent signals and by evaluating the ratio of the fluorescence intensities is possible to understand the probe distribution in the lipid network. The pyrene monomer fluorescent spectrum consists of five peaks. It is well established that the ratio I1/I3 between the intensities of the first (I1) and third (I3) vibration bands of the pyrene fluorescence spectrum (corresponding to 372 nm and 382 nm, respectively) is related to the polarity of the pyrene environment. Low values of the I1/I3 ratio correspond to a nonpolar environment. This ratio increases as the polarity of the medium rises^17^. Since pyrene is solubilised inside the hydrocarbon chain of the NE shell, the information obtained from fluorescence of pyrene in our systems refers to the lipophilic shell of NE pigeonhole^18^. The presence of pyrene intramolecular excimers depends on the rate of conformational change of the molecule, which is sensitive to the viscosity of the probe microenvironment^19^. Hence, the IE/IM ratio, where IM and IE stand for the intensity of the monomer and the excimer fluorescence, respectively, is used to estimate the microviscosity. Because of its high hydrophobicity, the solubilisation zone of pyrene occurs in the lipidic shell, as was established in the case of polymeric micellar solutions^20^. The pyrene probe may also evidence (only qualitatively) the micropolarity variation in the solubilisation region, by the change in the ratio of monomer vibronic band intensities measured at 377 nm and 397 nm^21^.

### Physicochemical stability

Specific studies on physical stability of NEs, composed of Tw20 and Neem oil at different ratios, were carried out to investigate whether significant size and ζ-potential changes in NE dispersion occur during storage at the two selected temperatures, using water or Hepes buffer as aqueous phase. The NEs were stored at 4 and 25 °C for a period of 90 days. Samples were analysed at definite time intervals (1, 30, 60 and 90 days) and the ζ-potential and the mean of hydrodynamic diameter of vesicles were measured as previously described.

Biological studies were carried out in the presence of simulated cerebrospinal fluid (CSF), pH 7.3, to evaluate the stability of NE if administered nasally. The CSF was prepared as described by McNay et al.^22^. NEs were diluted in CSF to obtain a final CSF concentration of 45% in Hepes buffer (pH 7.4, 10 mM). The average size, polydispersity index and ζ-potential were evaluated at different time points (30, 60, 120 and 180 min) at 37 °C.

### Small angle X-ray scattering (SAXS)

Small angle X-ray scattering (SAXS) experiments were carried out at the European Synchrotron Radiation Facility (ESRF, Grenoble, France). Measurements were acquired at the high-brilliance beamline ID02, with two sample-detector distances, in the region of momentum transfer 0.017 nm^−1^ ≤ *q* ≤ 5 nm^−1^ (*q* = (4*π*/*λ*) sin(*θ*/2), where *θ* is the scattering angle). Samples were inserted in 2 mm capillaries (KI-beam, ENKI, Concesio, Italy) and placed horizontally onto a thermostatted sample holder (*T* = 25 ± 0.1 °C). Very shorts frames were collected (exposure time 0.1 s) and checked for effects induced by radiation damage before be averaged. After radial integration and background subtraction, the SAXS profiles report the scattered radiation intensity as a function of momentum transfer, *q*. The intensity decay behaviour can give information on the structure of the particles in solution on the nanometre length scale. Synchrotron small-angle X-ray scattering (SAXS) techniques can give information on the internal structure of formulations for drug delivery^23^ ^,^ ^24^.

### Transmission electron microscopy (TEM)

Ten microlitres of diluted samples was adsorbed for 1 min onto Formvar-coated copper grids, then negatively stained with 2% filtered aqueous sodium phosphotungstate adjusted to pH 7.0. Negatively stained preparations were observed by a Philips 208 S transmission electron microscope (Philips Electron Optics, Eindhoven, The Netherlands) at 80 kV.

### Radical scavenging activity

#### Free radical scavenging activity (DPPH)

Test solution (1 ml) was added to DPPH solution (4 ml, 0.004% methanolic solution). The sample absorbance was noted at 517 nm after 30-min incubation at room temperature in the darkness. Results were expressed as milligrams of Trolox equivalents per sample amount (mg TEs)^25^.

#### ABTS (2,2 azino-bis (3-ethylbenzothiazoline-6-sulphonic acid)) radical cation scavenging activity

ABTS^+^ radical cation was produced directly by reacting 7 mM ABTS solution with 2.45 mM potassium persulphate and allowing the mixture to stand for 12–16 h in the darkness at the room temperature. Firstly, ABTS solution was diluted with methanol to an absorbance of 0.70 ± 0.02 at 734 nm. Test solution (1 ml) was mixed with ABTS solution (2 ml) and mixed. The sample absorbance was noted at 734 nm after 30-min incubation at room temperature. Results were expressed as milligrams of Trolox equivalents per sample amount (mg TEs)^26^.

#### Evaluation of total antioxidant capacity by phosphomolybdenum assay

Test solution (0.3 ml) was mixed with 3 ml of reagent solution (0.6 M sulphuric acid, 28 mM sodium phosphate and 4 mM ammonium molybdate). The sample absorbance was read at 695 nm after 90-min incubation at 95 °C. Results were expressed as millimoles of Trolox equivalents per sample amount (mmol TEs)^27^.

### Reducing power assays

#### Cupric ion reducing (CUPRAC) method

Test solution (0.5 ml) was added to reaction mixture containing CuCl_2_ (1 ml, 10 mM), neocuproine (1 ml, 7.5 mM) and NH_4_Ac buffer (1 ml, 1 M, pH 7.0). Similarly, a blank was prepared for each sample (sample solution (0.5 ml) and reaction mixture (3 ml) without CuCl_2_). Absorbance at 450 nm was read after 30-min incubation at room temperature. Results were expressed as milligrams of Trolox equivalents per sample amount (mg TEs)^28^.

#### Ferric reducing antioxidant power (FRAP) method

Sample solution (0.1 ml) was added to FRAP reagent (2 ml) containing acetate buffer (0.3 M, pH 3.6), 2,4,6-Tris(2-pyridyl)-*s*-triazine (TPTZ) (10 mM) in 40 mM HCl and ferric chloride (20 mM) in a ratio of 10:1:1 (v/v/v). Then, the absorbance was read at 593 nm after 30-min incubation at room temperature. Results were expressed as milligrams of Trolox equivalents per sample amount (mg TEs)^29^.

#### Metal chelating activity on ferrous ions

Test solution (2 ml) was added to FeCl_2_ solution (0.05 ml, 2 mM). The reaction was initiated by the addition of 5 mM ferrozine (0.2 ml). Similarly, a blank was prepared for each sample (sample solution (2 ml), FeCl_2_ solution (0.05 ml, 2 mM) and water (0.2 ml)). Then, the absorbance of sample and blank was noted at 562 nm after 10-min incubation at room temperature. Results were expressed as milligrams of EDTA equivalents per sample amount (EDTAEs)^30^.

#### Cell culture conditions and treatments of cells with NEs

HEp-2 laryngeal cancer cells were grown in MEM medium (Sigma), supplemented with 1% penicillin–streptomycin and 10% foetal calf serum (FCS) in a 5% CO_2_ atmosphere. Proliferation was evaluated by 3-(4,5-dimethylthiazol-2-yl)-2,5-diphenyltetrazolium bromide (MTT) assay as described^27^. Briefly, 96-well plates were seeded with 5 × 10^3^ cells/well and different amounts of Neem oil were added to the cells, either as NEs (A2 sample) or as free oil. After 24-h exposure of the cells to the different preparations, 20 μl of a 5 mg/ml MTT solution in PBS was added to each well, and the plates incubated for 3 h. After the formazan crystals were dissolved by the addition 100 μl of dimethyl sulphoxide, optical density (OD) at 570 nm was determined with a spectrophotometer/fluorimeter microplate reader (PerkinElmer, Hopkinton, MA)^31^ and the values measured for treated cells, compared to those of untreated controls.

To visualise the uptake of NEs by HEp-2 cells, NEs loaded with Nile red dye (NE-NR) were prepared. HEp-2 cells seeded on cover slips into a 12-well plate were exposed to either NE-NR (10 μL of sample A1 NE-NR in 1 ml of the cell culture medium) or to free NR prepared as 1 mg/mL stock solution in acetone and used at a final concentration of 100 ng/mL. After 7- or 24-h incubation, cells were washed with phosphate-buffered saline solution (PBS) and fixed in methanol/acetone (1:1) for 5 min at −20 °C. After three washes, coverslips were mounted on slides with 0.1% (*w*/*v*) p-phenylenediamine in 10% (v/v) PBS, 90% (v/v) glycerol, pH 8.0 and the specimens observed by fluorescence microscopy using a Leica DM4000 fluorescence microscope equipped with an FX 340 digital camera.

### Statistical methods

Statistical significance of differences was evaluated by one-way ANOVA with repeated measures followed by *post hoc* paired Student’s *t*-test.

## Results and discussion

The Neem oil nanoemulsions, prepared by adding the amphiphilic substance (Tw20) at different weight ratio, were obtained by the method previously described. The role of the surfactant in NE formation is to facilitate the stabilisation process^2^ ^,^ ^3^. Typical examples of aqueous soluble surfactants are nonionic surfactants (e.g. Tw20) which are preferred because they are usually less irritating than the ionic ones^32^.

The major feature of nanoemulsions is their great stability of droplet suspension. Stability of an emulsion is hampered in two ways: flocculation by coalescence and Ostwald ripening. In nanoemulsion systems, flocculation is naturally prevented by steric stabilisation, essentially due to the submicrometric droplet size. The second one is the reduction of the configurational entropy which occurs when interdroplet distance becomes lower than the adsorbed layer thickness. In case of the second problem, stability is dependent on the droplet radius r, the Hamaker constant A and the adsorbed layer thickness δ. The stability is very high if the δ/r value is high. In case of nanoemulsion droplets, δ/r becomes extremely high in comparison with macroemulsions, which completely prevents their capability to coagulate^2^.

The results reported in Table 1 for all NEs were part of the general factorial design to establish the combination of variables and their interactions yielding nanoemulsions with desirable properties for nose-to-brain targeting. For each dependent variable (hydrodynamic diameter, PDI and ζ-potential), the effects corresponding to the investigated factors (emulsifier amount and aqueous phase type) and interactions were taken into account.

To stabilise the NE droplets and prevent the formation of aggregates, NEs need to show an appropriate ζ-potential value able to avoid droplet coalescence or fusion by repulsive, steric or electrostatic effects^33^.

DLS measurements indicate that different surfactant ratios form NEs with different but comparable sizes (Table 1), but in general, it can be pointed out that increasing surfactant content, the dimensions decrease. The dimensions of NEs in water or Hepes buffer are the same for the same sample. Furthermore, all samples show negative ζ-potential values (Table 1), indicative of the absence of droplet aggregation. There is a significant difference in ζ-potential values, if NEs have water or Hepes buffer as external phase. This is probably due to the presence of salt and to the ion content. This content is greater in buffer solution, so in this solution, ions will be able to adsorb on NE droplet surface and so to increase ζ-potential absolute values.

As reported by Đorđević et al.^34^, at a given concentration of surfactants in the formulation, more surfactant molecules are expected to be adsorbed per unit area in case of larger droplets leading to greater negative charge and, hence, more negative ζ-potential values. It is obvious that all nanoemulsions prepared with different aqueous phase types show different ζ-potential values.

Apart from providing the hydrodynamic diameter and ζ-potential value, DLS provides also valuable information on the homogeneity of the suspension. A single sharp peak in the DLS profile implies the existence of a single population of scattering particles. The polydispersity index (PDI) values of NEs are reported in Table 1: it is clear that all samples are homogenous and possess NE population with limited dispersity (PDI < 0.3) and reduced dimensions useful to carry out a nose-to-brain administration. For the emerged reasons, only data related to samples with Hepes buffer (10^−2 ^M pH 7.4) as external phase will be presented and discussed, although every experiment has been performed for all the samples reported in Table 1.

In order to characterise the nature of the lipophilic shell, microviscosity and polarity have been evaluated. Pyrene-loaded NEs were prepared following the procedure described previously and the fluorescence spectrum allowed calculating the values of polarity and microviscosity reported in Table 1. Polarity and microviscosity values are quite similar for all NEs; this is probably due to the formation of the same shell and in particular of the same inner structure independently from the surfactant amount used in the preparation of NEs.

**Figure 1.**
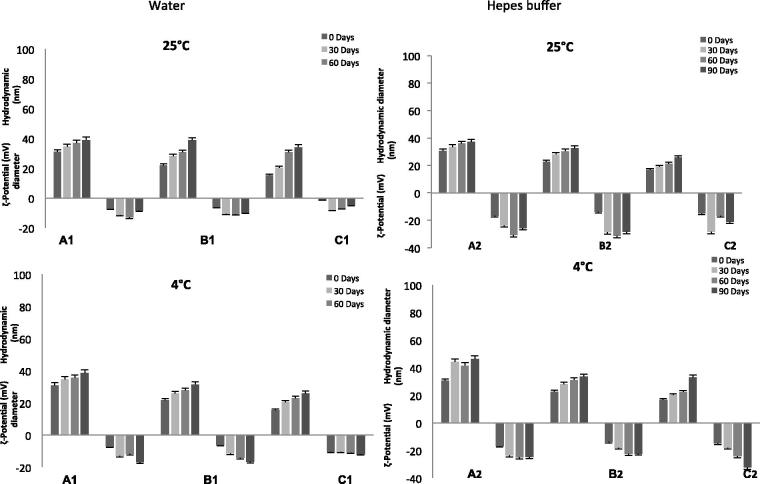
Shelf-life stability of evaluated NEs. Hydrodynamic diameter and ζ-potential values of different NEs (A–C) prepared using water^1^ or Hepes buffer^2^ as aqueous phase up to 90 days stored at 25 and 4 °C temperatures. Results are expressed as means ± SD (*n* = 3).

NEs stability is a complex issue and involves chemical, physical and biological stability, which are all interrelated. The evaluation of these parameters is fundamental to determine the potential *in vitro*/*in vivo* applications in nanomedicine. The physical stability studies of NEs are shown in Figure 1. From the analysis of reported results, it is clear that all NE samples, stored at the two different temperatures and prepared with the two different aqueous solutions, show high colloidal stability. In 90 days, dimension and ζ-potential variations of all NE samples at the two different temperatures show a similar trend, with no significant changes in physical characteristics of the formulation. Stability studies performed at 25 and 4 °C show that all analysed samples are stable for at least three months.

The effect of CSF on the stability of the NEs is another important aspect to take into account in order to evaluate and to understand the behaviour of the vesicles when they are in contact with fluids different from Hepes buffer. Experiments were performed at 37 °C checking the size and ζ-potential of the droplets by DLS analysis up to 3 h. During the time interval analysed, the same trend is observed for all NEs. NEs in 45% CSF maintained their size and ζ-potential values.

**Figure 2.**
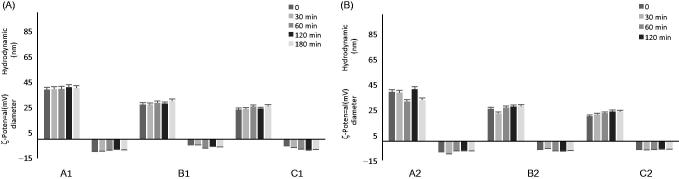
Stability in CSF. Size and ζ-potential measurements by DLS of different NEs (A–C) prepared using water (A) and Hepes buffer 10^−2 ^M, pH 7.4 (B) at 37 °C up to 3 h.

**Figure 3.**
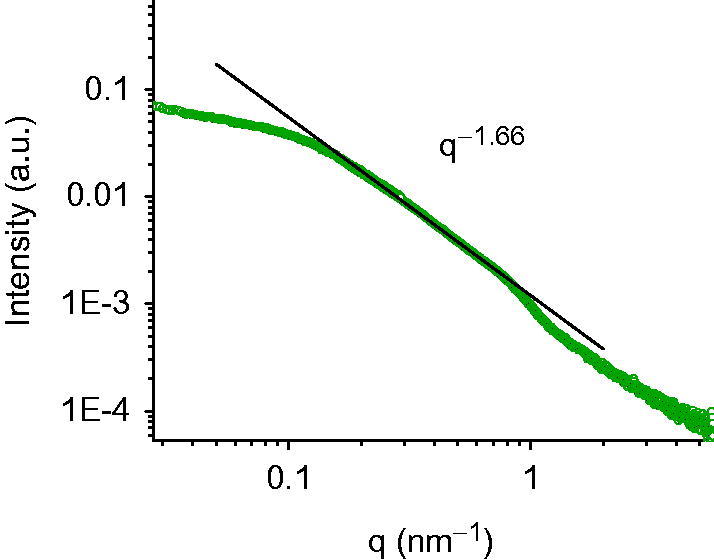
SAXS intensity spectrum. Scattered radiation intensity as a function of momentum transfer *q* of A2 sample at room temperature. The flattening of the intensity for *q* < 0.1 nm^−1^ shows that aggregates have a globular shape of finite size, of the order of 30 nm. At higher *q* (0.18 < *q* < 0.75 nm^−1^), the intensity *I*(*q*)/*q* ^−1.66^. This decay behaviour is characteristic for an internal core composed by connected substructures or sponge phases.

It can be concluded that in the presence of CSF, the selected samples show a good stability, with no significant changes in dimensions and ζ-potential values (Figure 2). X-ray scattering experiments have been performed to characterise the structure of nanoemulsions on the nanometre length scale. Figure 3 reports the SAXS intensity spectrum for A2. Structural features are identifiable in different regions of the spectrum; at low *q* (*q* < 0.1 nm^−1^, large lengths), the intensity profile shows that aggregates have a globular shape of finite size, of the order of 30 nm. At higher *q* values, in the region 0.18 < *q* < 0.75 nm^−1^, the intensity as a function of *q*, *I*(*q*), is proportional to *q* ^−1.66^. This decay behaviour, evidenced as a line of slope −1.66 in the log–log scale of Figure 3, is characteristic of connected substructures or sponge phases, on distances between 8 and 30 nm. Results show that the internal structure of the aggregates is not a classical core-shell structure with well-defined interfaces between the oil region, the surfactant region and the water. The oil phase is finely dispersed in nanosubstructures condensed by Tween molecules in a 30 nm aggregate.

At lower oil content, the overall dimensions are quite smaller, but with very similar internal structure (samples are less monodisperse). Similar oil substructures are confined in a smaller nanosized aggregate stabilised by a higher fraction of surfactant.

**Figure 4.**
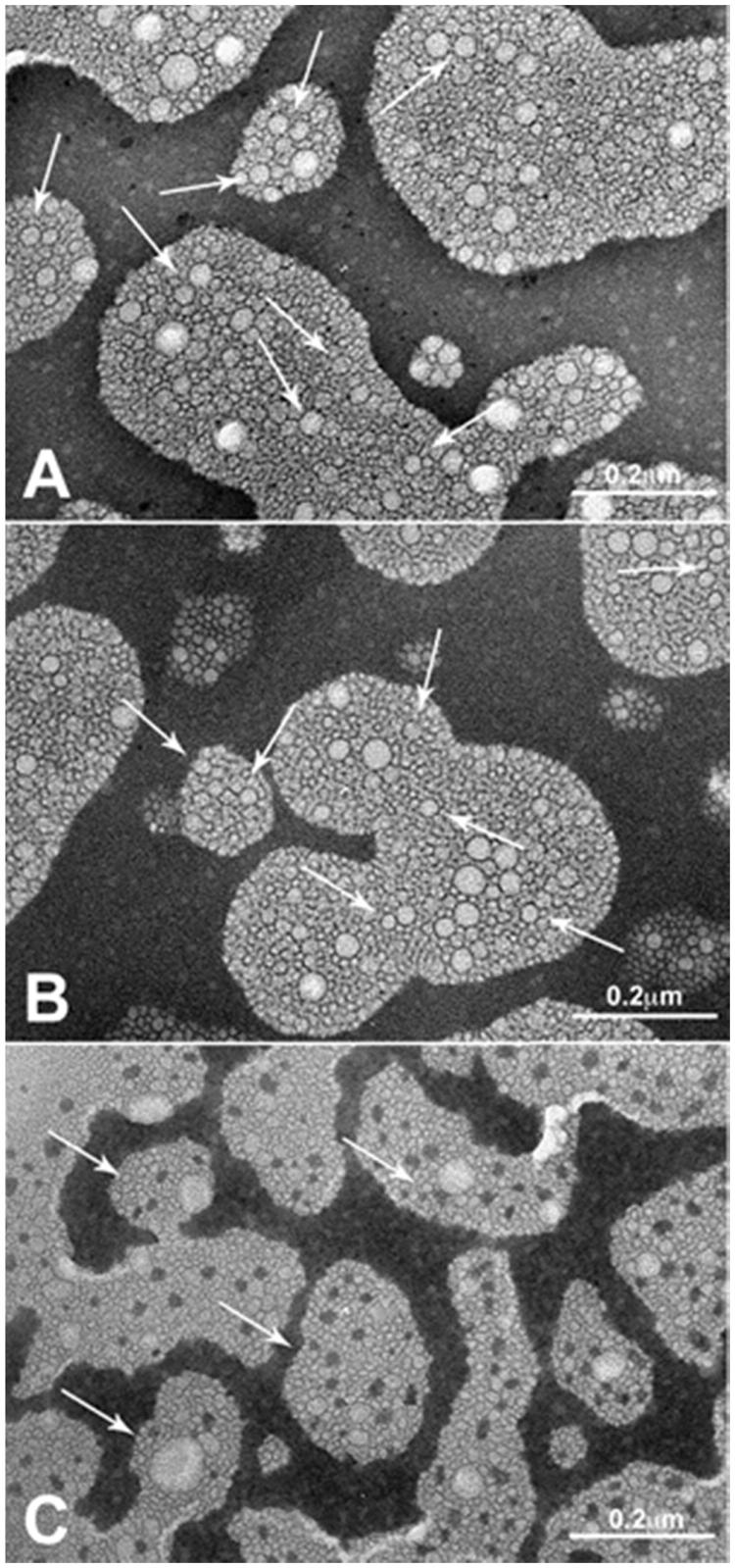
Transmission electron photomicrographs of the Hepes nanoemulsion samples. Panel A: A2; Panel B: B2; Panel C: C2. Arrows indicate NE sizes corresponding to DLS measures.

In order to investigate the morphology of the NEs, the three Hepes samples (A2, B2 and C2) were investigated by TEM. Figure 4 reveals that all nanoemulsion samples were almost spherical in shape, similar to other nanoemulsions prepared with different types of oil^35^. The TEM analyses confirm the presence of lipid emulsion droplets with an internal structure characterised by the presence of core with sponge-like features.

Starting from the promising biological activities of Neem oil, we firstly analysed the effect of this natural product in terms of antioxidant, reducing power and chelating properties. Pure Neem oil was characterised by a high antioxidant effect as demonstrated by a large number of assays with respect to reference compounds such as Trolox and EDTA (Table 2). These results also suggest that standardised Neem oil might be beneficial for the preparation of nanoemulsion with putative pharmacological effects.

**Table 2.** Antioxidant and chelating properties of pure Neem oil.

Assay	Results^a^
DPPH scavenging activity (mg TEs/g oil)	7.59 ± 0.07
ABTS scavenging activity (mg TEs/g oil)	14.78 ± 2.33
CUPRAC assay (mg TEs/g oil)	76.14 ± 5.57
FRAP assay (mg TEs/g oil)	51.13 ± 1.42
Phosphomolybdenum assay (mmol TEs/g oil)	0.77 ± 0.06
Metal chelating activity (mg EDTAEs/g oil)	19.26 ± 0.56

a Values expressed are means ± SD of three parallel measurements.

TE: Trolox equivalent; EDTAE: EDTA equivalent.

Moreover, the prepared nanoemulsions were subjected to the same biological evaluation as regards their antioxidant, reducing power and chelating properties. As shown in Table 3, antioxidant activity (ABTS and DPPH tests) was strongly influenced by the aqueous component, being Hepes buffer better than water. In the other assays (CUPRAC, FRAP, phosphomolybdenum and chelating tests), the activities were quite comparable without any significative difference between Hepes buffer and water. These biological results, in terms of Trolox or EDTA equivalents, were also not modified by the decreasing content of loaded Neem oil amount (from 9.2 mg/mL for A/A1 to 2.6 mg/mL for C/C1). Collectively, these results could suggest not only a structural role, but also a protective effect of Neem oil as a pivotal component for the stability, conservation and microbial resistance of these nanoemulsions.

**Table 3.** Antioxidant and chelating properties of prepared Neem oil-loaded NEs.

Samples	ABTS radical scavenging activity (mg TEs/L sample)	DPPH radical scavenging activity (mg TEs/L sample)	CUPRAC (mg TEs/L sample)	FRAP (mg TEs/L sample)	Phosphomolybdenum (mmol TEs/L sample)	Metal chelating activity (mg TEs/L sample)
A1	35.02 ± 0.47*	22.72 ± 0.43	55.94 ± 2.28	61.90 ± 0.02	2.49 ± 0.02	39.34 ± 0.14
A2	53.06 ± 1.87	87.79 ± 9.87	55.51 ± 3.37	62.76 ± 0.97	2.91 ± 0.01	43.35 ± 0.05
B1	35.55 ± 0.24	30.52 ± 3.12	56.65 ± 0.73	60.99 ± 2.90	2.48 ± 0.07	38.49 ± 0.22
B2	46.71 ± 1.66	90.92 ± 8.64	59.91 ± 0.52	70.81 ± 12.76	2.33 ± 0.28	42.60 ± 0.22
C1	33.36 ± 0.19	15.75 ± 1.62	52.92 ± 0.14	56.69 ± 4.87	2.87 ± 0.09	40.38 ± 0.44
C2	49.02 ± 4.73	83.77 ± 3.10	50.73 ± 3.10	58.26 ± 1.85	5.54 ± 1.85	43.45 ± 0.02

* Values expressed are means ± SD of three parallel measurements.

TE: Trolox equivalents; EDTAE: EDTA equivalents; Na: not active.

It has been reported that bioactive compounds extracted from Neem oil could affect multiple cellular pathways leading to apoptotic cell death^36^. Moreover, in different tumour cell lines, Neem oil has shown to exert anticancer activity via caspase-dependent and independent apoptosis as well as autophagy-mediated cell death^37^.

**Figure 5.**
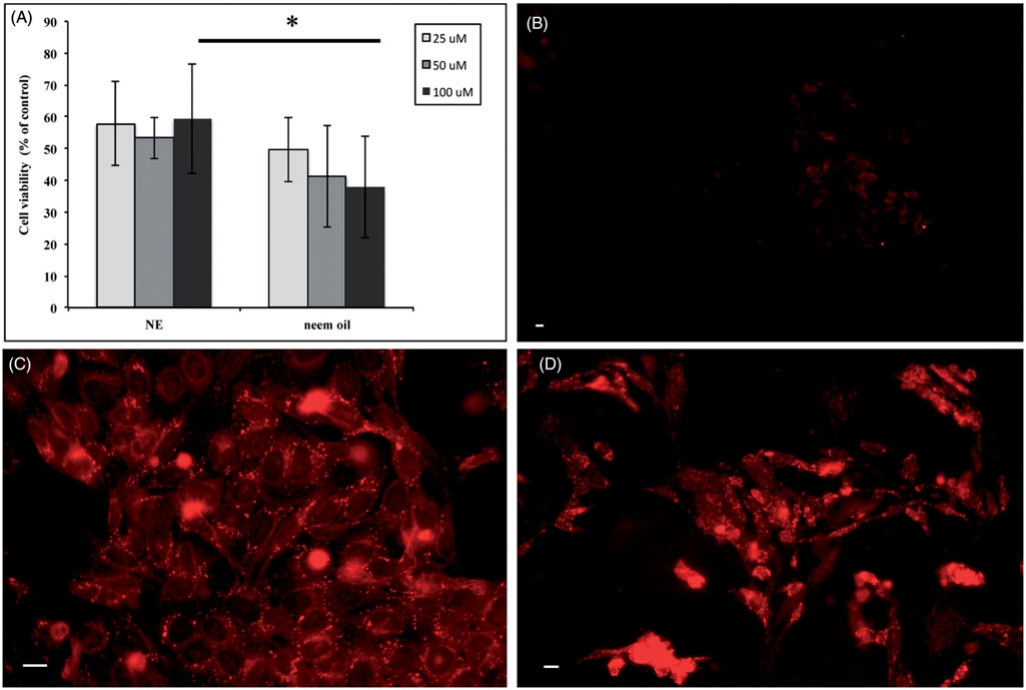
Exposure of HEp-2 cells to Neem oil-based NEs. HEp-2 cells were treated with NEs or free Neem oil as described (see Methods). The OD values obtained by MTT assay for treated cells were converted into numbers of cells on a standard curve and expressed as percentages of untreated controls. Bars represent the mean of three independent experiments ± SD. *p < .05 (Panel A). Fluorescence microscopy images of HEp-2 control cells exposed to free Nile red (Panel B) and HEp-2 cells exposed to Nile red-loaded NEs for 7 (Panel C) and 24 h (Panel D) obtained by fluorescence microscopy as described (see Methods). Scale bar: 10 μm.

In order to use NEs as carriers of pharmacological active compounds, we tested the effects of Neem-based NEs on the proliferation of tumour cell lines of different origin. Figure 5 illustrates the results obtained from HEp-2 human laryngeal carcinoma cells, treated for 24 h with increasing amounts of NEs. The bar graph (Panel A) shows that the proliferation of cells exposed to 100 μM Neem oil complexed into NEs is significantly higher than that detected in the samples treated with the same amount of free oil, indicating that the toxic effect of this agent is reduced in our oil/surfactant NE preparation.

To further evaluate the NEs as potential carriers of bioactive substances, we assessed their cellular uptake by fluorescence microscopy analysis of HEp-2 cells exposed for either 7 or 24 h to Nile red-loaded NEs or Nile red dye alone. No signal could be detected in HEp-2 control cells exposed to free Nile red for 7 h and similar results were obtained after 24 h (Panel B). In contrast, a strong fluorescent intracellular signal, as a punctuated pattern increasing with the time of incubation, could be visualised in the cytoplasm of cells exposed to the NEs (Panel C: 7 h and Panel D: 24 h). Altogether, these data strongly suggest that our preparations might represent suitable systems to deliver bioactive compounds into the cells.

## Conclusion

This study confirms the possibility of preparing NEs by Tween 20 and Neem oil at different weight ratios. Among the different formulation tested, the three (both in water and in Hepes buffer) prepared and characterised in the present research paper show the smallest hydrodynamic size and a homogenous sample size distribution. The evaluation of the antioxidant activity of Neem oil alone or as NEs confirms that the structured oil in NEs does not lose its activity and is less cytotoxic than the free oil after interaction with HEp-2 cells. A future step will be to load a drug inside NEs to enhance Neem oil antioxidant activity or to offer a double therapeutic effect in the same formulation. This type of system could be successfully applied in nose-to-brain delivery.
